# The Ganglionic Nervous System; Its Structure, Functions, and Diseases

**Published:** 1859-01-01

**Authors:** 


*The Ganglionic Nervous System; its Structure, Functions, and Diseases.* By JAMES GEORGE DAVEY, M.D. London. 1858.

"IT is the nature of an hypothesis," says Tristram Shandy, "when once a man has conceived it, that it assimilates everything to itself as proper nourishment; and, from the first moment of your begetting it, it generally grows stronger and stronger by everything you see, hear, read, or understand." We fear that the hypothesis set forth in the work which we are about to notice is but another illustration of this profound dictum. Dr. Davey is of opinion that the ganglionic nervous system, or, in other words, the sympathetic nervous system, is the head and front of all the functions of organic life. "If," he writes, "the *organic functions*, as they obtain in the 'animal,' are to be referred to the *ganglionic nervous system;* or, what is the same thing, to the *irritability* of which it is the immediate seat, it follows, admitting the existence or operation of the same 'organic functions' in the vegetable kingdom, that plants are necessarily endowed with nerve-structure, and that *this* constitutes the *analogue* of the *ganglionic nervous system* in man and the higher animals."—(p. 4.)

This sweeping corollary fittingly paves the way for the observations which Dr. Davey conceives warrant the conclusion, that the organic functions, as witnessed in the animal economy, are to be referred to the ganglionic nervous system.

As to the structure of this system, he arrives at the following conclusions:—

"*a.* The ganglionic nervous system first exists in a molecular form; and that it is made up of globules dispersed throughout the homogeneous texture of the animal, as in the acriti, the lower Entozoa, &c.; as such it has also been presumed to exist in the early human embryo.

"*b.* These nervous globules, arranging themselves in a longitudinal series, form filaments, and these 'filaments' at length form a central point, a ganglion (*solar ganglion,* Anderson).

"*c.* Ascending the scale of animal creation, and arriving at the *Mollusca,* nervous matter is found secreted about and around the œsophagus, and on the dorsal aspect of the animal (supra-œsophageal ganglion); this nervous matter becomes, in the higher animals and in man, the tubercula quadrigemina or medulla oblongata.

"*d.* Still ascending, the cyclo-ganglionic nervous system becomes more complicated, whilst the several tissues and structures subordinate to the same, including the cerebro-spinal system, are proportionately amplified; the increased development of the former, and the general augmentation and addition of parts in the latter, being in the relation to each other as *cause* and *effect*." (pp. 65, 66.)

Now, although we must confess to an inability to comprehend, from the text, the precise meaning of the ganglionic nervous system first existing in a molecular form, as affirmed in segment *a* of the conclusions; or to solve the paradox of a globular nervous system co-existing with homogeneity of texture in an animal; we cannot remain in doubt as to the signification which Dr. Davey desires to be attached to the presumption respecting the existence of the ganglionic nervous system in the early human embryo. Dr. Davey writes, *àpropos* of Dr. Carpenter's views on cell-growth:—

"Now, if we will be at the trouble to inquire more particularly into these 'cells' so constantly mentioned by Dr. Carpenter; if we will take the pains to investigate the nature of their vital properties, their specific offices in the animal economy, we shall, I think, conclude presently that the aforesaid *cells* are nothing more or less than the 'nervous globules' (the seat or source of 'nervous sensibility,'—Anderson), distributed in the homogeneous structures of the Polygastrica and Polypifera, &c., and found to constitute the very sum and substance of the early embryo (human). What says Dr. Carpenter then of the 'cells,' *alias* 'nervous globules?' At page 87, we find his evidence in favour of the identity here mentioned thus expressed—viz.: 'It seems to have been established, as the aggregate result of the labours of many observers, that in animals as in plants, all the parts in which active *vital* changes are taking place essentially consist of cells, which may be regarded *as the real instruments* of these operations.' At page 103 there occur these words: 'Still we shall find the general rule to hold good, that all the animal tissues are developed in the first instance through the medium of cell-life; and that in the organs subservient to the strictly *vital* operations, *cells* remain the essential *instruments*.'

"The inevitable conclusion from the facts and reasoning of Dr. Carpenter is, that the organic or vegetable functions, as observed in even plants, and in the lowest forms of animal life, are due to 'vital operations' consequent on their 'cellular type of structure.'

"I cannot myself doubt that nervous matter *is* present in 'a diffused form,' *i. e.*, incorporated with the other tissues, in all those animals wherein no *distinct* nerve structures have been made out. I cannot dissociate the positive *vitality* of such animals from a highly elaborated (imperceptible?) nervous organism. There seems every reason to believe that the 'cell-life' mentioned by Dr. Carpenter is, in all respects, identical with the 'impetum faciens' of Hippocrates, the 'materia vitæ' of Hunter, the 'nisus formativus' of Blumenbach, the 'irritability' (*motions without force*) of Haller, the 'creative force' of Muller, and so on." (pp. 58, 59.)

To confound, as has been done in the paragraphs quoted, the general doctrines of cell-growth and the nature of cells with the attributes and properties of certain cells having a special function, and which are stated to exist in what are nevertheless asserted to be homogeneous structures; and, also, with the cells which constitute the human embryo in its earlier stages of development, is to reduce the whole matter to verbiage, of which the meaning entirely escapes our comprehension.

It is needless to examine the remaining conclusions of Dr. Davey on the structure of the ganglionic system, as they are based more upon the opinions quoted above on the nature of cells than upon actual examination.

Dr. Davey is scarcely happier in his speculations upon the functions, than he is upon the structure of the ganglionic nervous system. He considers that it "performs" the whole of the organic functions (those which give us "the notion of life"), excretion, nutrition, exhalation, absorption, calorification, &c.; that it presides over the brain and spinal cord; and that it is the source of *instinct*; in short, that the ganglionic nervous system is the source and mainspring of all the vital actions of *an* organism.

It is not always easy to follow the course of the argument by which Dr. Davey arrives at this startling generalization. The existence of "irritability" in an animal from which the brain and spinal marrow have been removed, or in a limb which, as in the experiments of Sir B. Brodie, is left attached to the trunk by the blood-vessels only, he considers as being dependent upon the ganglionic nervous system (pp. 74-77); but he advances no argument in support of this conclusion other than his preconceived notions of the supreme importance of the system in the animal economy. Not only is the ganglionic system regarded as the cause of "irritability," but it possesses itself the said "irritability,"—whatever that may be, for in one place Dr. Davey uses the term as synonymous with vitality (p. 74); in another as equivalent to contractility (p. 77); in a third it becomes "a peculiar kind of sensibility" (p. 79); and in a fourth it represents the "true spinal phenomena" (p. 75). Concerning this phenomenon, "irritability," Dr. Davey writes:—

"I have on many occasions performed the experiment on the frog, as detailed by Dr. M. Hall, and I have invariably found that the removal of the viscera and ganglionic system, as described by him, is fatal to the life of the animal; and that what Dr. M. Hall has called the 'true spinal phenomena' (irritability) as manifested by the eviscerated and headless being, are continued no longer than *that peculiar kind of sensibility* (contractility) which may be at any time observed in the heart or intestinal canal, and so on, of any animal, after the removal of either from the trunk. That the vitality of the spinal cord—and not less its capacity to execute its normal functions in the animal economy—is derived from the ganglionic nervous system, is to my mind certain, and not less easy of demonstration than is the presence of the sun at noonday.

"One word more, ere I conclude my criticisms of the experiments of Drs. le Gallois and Hall. The latter gentleman finishes the description of his famous experiment on the frog with these few words—viz., 'Having thus, then, clearly laid before you the distinction which I wish to insist upon—namely, that there is not a division of the nervous system into two parts only, but into three, pervading all the different parts of the whole animal frame—I shall venture to term them the cerebral, the true spinal, and the ganglionic systems.' I would remark that, whilst accepting this division of the nervous organism, I would submit that both the first and the second named are dependent on the third; that they derive not only their very existence and integrity from it, but also perform their respective functions in virtue only of the influence they receive from it; and that they are, as it were, employed by it to establish our dependency on, and relationship to, the external world, of which man forms a part." (pp. 79, 80.)

And further, Dr. Davey writes:—

"The cerebral and spinal systems of nerves together perform the *animal functions*, which, in the words of an eminent physiologist (Blumenbach), prove us feeling, thinking, and willing beings; they are the actions of the senses, which receive impressions; of the brain which perceives them, reflects upon them, and wills; of the voluntary muscles, which execute the will in regard to motion; and of the nerves, which are the agents of transmission; the brain is their central organ. The ganglionic system of nerves, with the solar ganglion for its central organ, performs the vital or organic functions—these are altogether independent of mind, and give us simply the notion of *life*. Secretion, nutrition, exhalation, absorption, and calorification, &c., being under its immediate influence and control throughout the whole body, it must preside equally over the *brain* as the stomach, equally over the *spinal cord* as the liver or uterus. In point of fact, if either one of these organs or viscera just named were removed from the influence of the ganglionic nerves entering so largely into its very composition, its specific vitality must cease—*i.e.*, the function it was wont to exercise would end in the individual—its contribution to the sum total of *life* would be withheld." (pp. 80, 81.)

Having thus exhausted the relation and asserted the supremacy of the functions of the ganglionic nervous system in organic life, it becomes necessary to ascertain the relation of that system itself to vital laws. Assuming, therefore (as Dr. Davey does), "for argument's sake," that there is such a thing as an "organizing principle," or "creative force" (p. 81), and "granting, for the sake of argument, that the *ganglionic* nervous system is the source and origin of this *power*, or *organizing principle*, or *creative power*" (p. 84), whence did the system receive its being? Dr. Davey at first asserts that it is not in the power of man to offer anything more than a very general reply to this query; but, bold of heart, he struggles through the *nifflheim* of the nisus formativus, and having previously written that "Even if the result of life—the functions of a part—should be called its life, life could not be said to be the result of organization, but of a power to which organization is an instrument" (p. 83); he arrives at the conclusion that life is a product of the nervous system.

"In considering the effects of certain vivisections on animals, as well as the consequences of accident to man, in so far as both these involve more immediately the ganglionic nervous system, the attention is with much certainty arrested by the fact that in the *annelida* 'their division into separate fragments does not destroy the organism, but, on the contrary, gives rise to the production of several distinct beings.' This circumstance is, in itself, highly interesting, and demonstrates to us the fact that the animal tissues (organism) at this particular stage of development are sustained in their integrity equally by the several *ganglia* forming a part of the ganglionic nervous tissue; whilst in man, including the more perfect animals, the vital principle (the '*anima*') is *in great part* generated in a central organ (the *solar ganglion*), and from it diffused through the whole body.

"It is rightly inferred that *life*, regarded in the abstract, is identical throughout all animate nature; in every genus, species, and variety; and in each individual of all three of these artificial divisions, 'life' is seen, or rather *known*, in connexion only with an especial nervous apparatus; and that these stand in relation to each other as *cause* and *effect*, it would seem impossible to doubt."—(pp. 105, 106.)

Here, then, we have the assertions that organization is the result of life, and that life is the result of organization; *i. e.*, life is the cause of life—the cause of the cause—a *reductio ad absurdum.*

We need not dwell upon the pathological reasons with which Dr. Davey seeks to support the foregoing propositions, nor detail the facts which he conceives demonstrate the truthfulness of his opinion, "that both the ordinary *nutrition* and, what is more, the *reproduction*, equally with the decline (atrophy) or decay of the organism (the very converse of the nutrient process), must be attributed only to the operations of the organic or ganglionic nervous systems," &c. (p. 114); neither need we do more than refer to his assertion, "that the successive increase of parts above the *medulla oblongata* is attributable to the operation of a preliminary cause—viz., the 'formative power,' or the ganglia of the sympathetic, *the germ of all animal life, whether cerebral, spinal, or organic*" (p. 116). It is simply requisite to direct attention to the transformation of the "formative power" into the ganglia of the sympathetic, in the preceding quotation, and to mention the subsequent conversion of the "*nisus formativus*" into the "*solar-ganglion*, including the ganglia of the great sympathetic" (p. 127). We may not touch upon the development of the argument by which Dr. Davey seeks to show that secretion, nutrition, and animal heat, have their source in the ganglionic system, and it is only necessary to quote the statements that—

"The *storge* is an *instinctive act*, peculiar to birds, &c.; and the great *animal heat* which attends it affords reason to conclude, that both instinct and animal heat have one common cause or origin." (p. 127, *note.*) . . . . . . "The presence of instinct is, it appears to me, an indication of the activity of the organic functions—*instinct* is one of these; it is always seen in combination with them; it partakes of their activity or otherwise. Instinct is ever manifested in proportion to the development of the organic nervous system; and, inversely, to that of the cerebro-spinal system. It is the substitute for intelligence, for reason. Instinct is to the wasp or beaver what experience (reason) is to man. Instinct prompts the idiot to the same automatic movements which are directed to the supply of its physical wants, as experience and reason may and do a healthy and sane man to eat, or sleep, or walk, and so on."—(pp. 135, 136.)

Moreover, we may only quote, without attempting to ascertain their signification, certain conclusions which, with other conclusions, are attached to a paper of considerable length on the excito-motory or diastaltic nervous system, and added to the physiological division of the work:—

"That both the external and internal excito-motory phenomena are alike dependent on the vital stimulus imparted by the great sympathetic nerve; and, in the former instance, through the instrumentality or medium of the medulla spinalis.

"That such is the relationship between the great nervous centres, viz., the brain, spinal cord, and the solar ganglion, that the second (viz., the spinal cord), being at one time under the dominion of the first, *i. e.*, the brain, and at another time under that of the third, *i. e.*, the solar ganglion, and being thus intermediate, will now manifest a cerebral or voluntary power (and this the result of consciousness, and, of course, volition), and then (the antecedent circumstances being dissimilar) display one of an involuntary, automatic, or instinctive, or excito-motory character; but this only, of course, when the organic nervous apparatus is in the ascendant, and the brain comparatively quiescent—or, in other words, when sensation (spinal) and motion, and not consciousness and volition (cerebral attributes) are in operation; if I may be allowed so to express myself.

"That the great sympathetic nerve is endowed with a specific and independent power, to which both the brain and spinal cord, and with them, of course, their functions are subordinate."

Dr. Davey's views on the pathology of the ganglionic nervous system, and the treatment of its diseases, are founded upon his physiological notions, and the vices of the latter prevail also in the former.

The purely speculative character of the majority of Dr. Davey's opinions renders it unnecessary that we should discuss the questions which he broaches; and the reasons which have determined physiologists to adopt the opinions which Dr. Davey seeks to controvert, are detailed at length in every standard work on physiology.

---

